# Investigating exchange, structural disorder, and restriction in gray matter via water and metabolites diffusivity and kurtosis time-dependence

**DOI:** 10.1162/imag_a_00123

**Published:** 2024-04-05

**Authors:** Eloïse Mougel, Julien Valette, Marco Palombo

**Affiliations:** Laboratoire des Maladies Neurodégénératives, Molecular Imaging Research Center (MIRCen), Université Paris-Saclay, Commissariat à l’Energie Atomique et aux Energies Alternatives (CEA), Centre National de la Recherche Scientifique (CNRS), Fontenay-aux-Roses, France; School of Psychology, Cardiff University Brain Research Imaging Centre (CUBRIC), Cardiff University, Cardiff, United Kingdom; School of Computer Science and Informatics, Cardiff University, Cardiff, United Kingdom

**Keywords:** dw-mrs, microstructure, exchange, cell membrane permeability, neurons, astrocytes

## Abstract

Water diffusion-weighted MRI is a very powerful tool for probing tissue microstructure, butdisentangling the contribution of compartment-specific structural disorder from cellularrestriction and inter-compartment exchange remains an open challenge. In this work, we usediffusion-weighted MR spectroscopy (dMRS) of water and metabolite as a function of diffusiontime*in vivo*in mouse gray matter to shed light on: i) which of theseconcomitant mechanisms (structural disorder, restriction, and exchange) dominates the MRmeasurements and ii) with which specific signature. We report the diffusion time-dependence ofwater with excellent SNR conditions as provided by dMRS, up to a very long diffusion time (500ms). Water kurtosis decreases with increasing diffusion time, showing the concomitant influenceof both structural disorder and exchange. However, despite the excellent experimentalconditions, we were not able to clearly identify the nature of the structural disorder (i.e.,1D*versus*2D/3D short-range disorder). Measurements of purely intracellularmetabolites diffusion time-dependence (up to 500 ms) show opposite behavior to water, withmetabolites kurtosis increasing as a function of diffusion time. We show that this is asignature of diffusion restricted in the intracellular space, from which cellularmicrostructural features such as soma’s and cell projections’ size can beestimated. Finally, by comparing water and metabolite diffusion time-dependencies, we attemptto disentangle the effect of intra/extracellular exchange and structural disorder of theextracellular space (both impacting water diffusion only). Our results suggest a relativelyshort intra/extracellular exchange time (~1-50 ms) and short-range disorder (still unclear if1D or 2D/3D) most likely coming from the extracellular compartment. This work provides novelinsights to help interpret water diffusion-time dependent measurements in terms of theunderlying microstructure of gray matter and suggests that diffusion-time dependentmeasurements of intracellular metabolites may offer a new way to quantify microstructuralrestrictions in gray matter.

## 1 Introduction

Diffusion-weighted MRI (dMRI) is a valuable radiological tool to non-invasively quantify thebrain structure at the cellular scale, the so-called*microstructure*(Alexander et al., 2019;Jelescuet al., 2020;Novikov et al., 2019). Among thedifferent dMRI approaches investigated in the past decades, measurements of the diffusion-time(*t_d_*) dependent dMRI signal have been shown to provide informationon the brain tissue inter-compartmental exchange (Hill et al.,2021;Jelescu et al., 2022;Nedjati-Gilani et al., 2017;Nilsson etal., 2009;Olesen et al., 2022;Stanisz et al., 1997;Zhang et al.,2021), structural disorder (Novikov et al., 2014;Palombo et al., 2013;Palombo, Ligneul, et al., 2018), and the restricted environment where the moleculesdiffuse over ~1-100 µm (Assaf & Cohen, 2000;Does et al., 2003;Drobnjak et al., 2010;Jespersen et al., 2018;Palombo et al., 2016;Panagiotaki et al., 2012).

In particular, it has been shown that measurements of the*t_d_*-dependence of water diffusivity (Aggarwal et al., 2012;Arbabi et al., 2020;Baron & Beaulieu, 2014;Does et al., 2003;Wu et al., 2014),D_W_(*t_d_*), and kurtosis (Aggarwal et al., 2020;Lee et al., 2020;Pyatigorskaya et al., 2014;Wuet al., 2018), K_W_(*t_d_*), provide uniquequantitative insight into these tissue properties. Varying the*t_d_*allows probing different length scales and thus assessing the spatial heterogeneity of thecellular microenvironment. For instance, at short*t_d_*, obtained withthe oscillating gradient spin echo (OGSE) sequence, a large D_W_variation as afunction of the oscillating-gradient frequency (inversely proportional to*t_d_*) was measured in cell-dense regions compared to other regions ofthe mouse brain, which is thought to reflect, among other things, restricted diffusion on alength scale of a few micrometers, thus in the order of the cell nucleus size (Aggarwal et al., 2012). At longer*t_d_*, whichcan be probed using, for example, pulsed gradient spin echo (PGSE), the length scales probed aremore on the order of tens of micrometers. For such*t_d_*, D_W_and K_W_are sensitive to demyelination in white matter (Aggarwal et al., 2020) or to 1D structural disorders associated with neurites in graymatter (GM) (Lee et al., 2020;Novikov et al., 2014), but it has also been shown to be influenced byexchange (Jelescu et al., 2022;H. Li et al., 2017;Olesen et al.,2022;Yang et al., 2018), and disentangling thedifferent contributions remains complex. Most importantly, some exchange regimes can alsoinfluence K_W_(*t_d_*), as shown by numerical simulations(Aggarwal et al., 2020): increasing the diffusion timewill result in increased K_W_until*t_d_*approaches acharacteristic time corresponding to a diffusion length comparable to the typical (permeable)restriction size; for longer*t_d_*, K_W_will then decreasedue to exchange, with a slope which is steeper as exchange gets faster (i.e., higherpermeability). However, as valuable as these simulations are, they do not fully mirror the truestructural complexity of the GM tissue, in particular the structural disorder of the intra- andextracellular space. Disentangling compartment-specific structural disorder frominter-compartment exchange, either experimentally or in simulations, remains verychallenging.

In contrast and complementary to water, diffusion measurements of*purelyintracellular*metabolites by diffusion-weighted MRS (dMRS) offer information about thebrain microstructure that is specific to the intracellular space and is not affected byinter-compartmental exchange (Assaf & Cohen, 1999;Palombo, Shemesh, et al., 2018;Ronen & Valette, 2015). Some brain metabolites are alsopreferentially compartmentalized within neurons, such as N-acetyl aspartate (NAA), and glialcells, such as myo-inositol (Ins), providing a probe of neuronal or glial structure, and can,for example, be used to estimate neurite radii and intracellular diffusivity (Ligneul et al., 2017;Palombo, Shemesh,et al., 2018). Interestingly,*t_d_*-dependent dMRS is anefficient tool for probing complex GM cell microstructure, as it was shown that dMRS issensitive, for example, to spines and leaflets density (Palombo,Ligneul, et al., 2018) and cellular processes branching (Palombo et al., 2016). Specifically,*t_d_*-dependence ofmetabolite diffusivity D_M_(*t_d_*) and kurtosisK_M_(*t_d_*) is expected to be influenced by features of thecellular structure at different length scales, but without influence of extracellular space orexchange with extracellular space (Ianus et al., 2021).Micrometric restriction (soma and projection size) or exchanges between soma and projection(Ianus et al., 2021) could be all specific informationof the intracellular environment. D_M_(*t_d_*) andK_M_(*t_d_*) would thus provide valuable and complementaryinformation to water to investigate GM. By comparing the*t_d_*-dependent diffusivity,D_M_(*t_d_*), and kurtosis,K_M_(*t_d_*), of intracellular metabolites withD_W_(*t_d_*)-K_W_(*t_d_*)one can potentially separate and quantify the relevant mechanism(s) driving each*t_d_*-dependency in GM.

The aim of the present work is to shed some light on the role of exchange, structural disorder, and restriction in GM, by exploiting the complementary information of D _W_ ( *t _d_* )-K _W_ ( *t _d_* ) and D _M_ ( *t _d_* )-K _M_ ( *t _d_* ) measured by dMRS. More specifically, it aims at:

(I) revisiting water diffusion time-dependenceD_W_(*t_d_*)-K_W_(*t_d_*)with excellent SNR conditions as provided by dMRS (i.e., measured in a large volume) and upto very long*t_d_*(500 ms)
(II) reporting for the first time, to our knowledge, intracellularmetabolite diffusion time-dependenceD_M_(*t_d_*)-K_M_(*t_d_*)up to very long*t_d_*(500 ms) to determine the signature ofintracellular diffusion
(III) combining results from (I) and (II) to try to disentangle theeffect of exchange and the signature of the extracellular diffusion.

## 2 Material and Methods

### 2.1 dMRS acquisition

#### 2.1.1 Ethics approval, scanner, and sequence

All experimental protocols were reviewed and approved by the local ethics committee (CETEAN°44), and authorized by the French Ministry of Education and Research. They wereperformed in an approved facility (authorization #B92-032-02), in strict accordance withrecommendations of the European Union (2010-63/EEC). All efforts were made to minimize animalsuffering, and animal care was supervised by veterinarians and animal technicians. Mice werehoused under standard environmental conditions (12-hour light-dark cycle, temperature: 22± 1°C and humidity: 50%) with*ad libitum*access to food andwater.

Experiments were performed on an 11.7 T BioSpec Bruker scanner interfaced to PV6.0.1(Bruker, Ettlingen, Germany). A quadrature surface cryoprobe (Bruker, Ettlingen, Germany) wasused for transmission and reception. Wild-type C57BL/6 mice were anesthetized with ~1.6%isoflurane and maintained on a stereotaxic bed with one bite and two ear bars. Throughout theexperiment, body temperature was monitored and maintained at ~36°C by warm watercirculation. Breathing frequency was monitored using PC – SAM software (Small AnimalInstruments, Inc., Stony Brook, NY). The sequence used for all acquisition was adiffusion-weighted stimulated-echo sequence (Ligneul et al.,2017) followed by a LASER localization (STE-LASER). Echo time was set to TE =33.4 ms (including the 25-ms LASER echo time) and was held constant for each acquisition.Repetition time was set to TR = 2500 ms. The duration of the pulsed diffusion gradientwas set to δ = 3 ms. Diffusion gradients were separated by the delay ∆,which was varied according to the protocol described in the next two sections. In addition, aVAPOR water-suppression module supplemented by an additional 21-ms hermite inversion pulseinserted during the mixing time was used for metabolite acquisition, respectively.

#### 2.1.2 Water acquisition

Water diffusion was measured in four mice. A spectroscopic volume of interest (1.5 ×0.8 × 2 mm^3^= 2.4 µL) was placed in the hippocampus, in such away that it consisted almost exclusively of gray matter (no visible white matter, and CSFcontamination <<1%). For each delay = 21.8, 31, 43.5, 101, 251, 501 ms,that is, resulting respectively in diffusion time*t_d_*=20.8, 30, 42.5, 100, 250, 500 ms, attenuation was measured for*b*=0.2, 0.7, 1.2, 2.0, 2.5 ms/µm² along one fixed direction, with 16repetitions.

#### 2.1.3 Intracellular metabolites acquisition

Metabolite diffusion was measured in seven mice. A spectroscopic voxel (7 × 1.5× 3 mm^3^= 31.5 µL) covering the two hemispheres was centeredin the hippocampus so that CSF contamination was minimal. Voxel composition (gray matter~94%,white matter~5%, and CSF~1%) was estimated a posteriori on anatomical images acquired with aRARE sequence with TE/TR=30/2500 ms, 78.1-µm isotropic resolution, and 0.5-mmslice thickness, using manual segmentation with the Fiji distribution of the ImageJ software,in the whole volume. For each delay = 43.5, 101, 251, 501 ms, that is, resultingrespectively in diffusion time*t_d_*= 42.5, 100, 250, 500 ms,attenuations of N-acetylaspartate (NAA), choline compounds (tCho), creatine +phosphocreatine (tCr), inositol (Ins), and taurine (Tau) were measured for*b*= 0.2, 1.0, 2.0, 3.2, 4.5, 6.0, 8.0 ms/µm² along one direction. Twoblocks of 32 repetitions interleaved between each*t_d_*and*b*value were acquired for*t_d_*= 42.5, 100,250 ms and three blocks of 32 repetitions were acquired to improve the SNR for*t_d_*= 500 ms.

### 2.2 Data processing and analysis

#### 2.2.1 Data processing

Individual scans were frequency- and phased-corrected before averaging. For wateracquisitions, the integral of water peak was computed on a 0.5-ppm window centered on the peakmaximum at each*b*and*t_d_*. For metabolites, eachspectrum was analyzed with LCModel (Provencher, 2001).Experimental macromolecule (MM) spectra, acquired for each*t_d_*using a double inversion recovery module (TI_1_= 2200 ms and TI_2_= 770 ms), at*b*= 10 ms/µm², were included inLCModel basis-sets containing simulated metabolite spectra.

#### 2.2.2 Data analysis

Remaining within these predefined*b*-value ranges (according to theconvergence radius of the cumulant expansion (Frøhlichet al., 2006) for one hand water and other hand metabolites, which exhibit about fourtimes slower diffusivity than water), for each*t_d_*, thediffusion-weighted signal S as a function of*b*was fitted with a non-linearleast-square method implemented in Matlab’s “lsqcurvefit” function toestimate the*t_d_*-dependent apparent diffusivity of water ormetabolite (D_W/M_(*t_d_*)) and kurtosis(K_W/M_(*t_d_*)), usingequation 1(Jensen & Helpern, 2003),for each*t_d_*.

$$S(b)=S(0)\times exp(-bD+1/6Kb^{2}D^{2})$$ [1]

## 3 Results & Discussion

### 3.1. Revisiting water diffusion time-dependence: which power law describes water t _d_ dependence?

#### 3.1.1 Methodological considerations

**Fig. 1.**
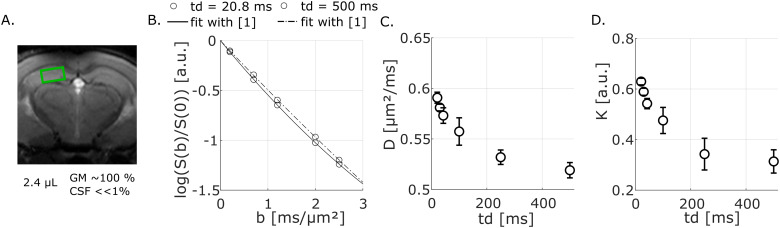
Water signal data. (A) Voxel of interest, for water acquisition. (B) Example of watersignal attenuation acquired at two different*t_d_*on one mouse andfits with the kurtosis representation [1] (lines). (C) Mean diffusivity and standard errorof the mean over four mice. (D) Mean kurtosis and standard error of the mean over fourmice.

The STE-LASER sequence provides a good water signal even at a long diffusion time and a high*b*value, allowing D_W_and K_W_to be evaluated over awide range of diffusion times [20.8; 500] ms and reaching a fairly long diffusion time(*t_d_*~500 ms), longer than in previous studies using PGSE sequence(Jelescu et al., 2022;Lee et al., 2020). With respect to attenuation (Fig.1B), the curvature of signal attenuation clearly attenuates with increasing diffusiontime, approaching but not reaching near mono-exponential diffusion at long diffusion time(K~0.3). The model given byequation [1]fits theattenuation of water well (as shown for one mouse onFig.1B), and this protocol provides a small inter-animal distribution of diffusionparameters (D_W_and K_W_), with a standard error of the mean between miceof 2% at shortest*t_d_*and ~15% at longest*t_d_*for D_W_and ~2% for K_W_.

Regardless of the precise time-dependence, average D_W_estimated in this study arein the same order of magnitude as ADC measured in rat (Jelescuet al., 2022) and mouse GM (Aggarwal et al.,2020;Zhang et al., 2021), and slightly lowerthan the ADC in human brain (Lee et al., 2020).K_W_is also in the same order of magnitude as in previous studies (Jelescu et al., 2022;Lee et al.,2020). This spectroscopic acquisition using the STE-LASER sequence is therefore ingood agreement with the literature.

#### 3.1.2 D _W_ and K _W_ decrease at increasing t _d_

Both D_W_and K_W_are time-dependent (Fig.1.C-D), over the whole*t_d_*range. D_W_decreasessignificantly (~12% difference with an F-test (F(5, 15) = 31.102,*p*< 0.001) for a Repeated-Measure ANOVA), reaches a plateau ~0.5 µm²/ms,when D_W_tends to the tortuosity asymptote (Fig.1C). K_W_also decreases (~50% with an F-test (F(5, 15) = 13.321, p< 0.001) for a Repeated-Measure ANOVA) and approaches ~0.3 (Fig. 1D), as the diffusion time increases, consistent with the diffusion notbeing Gaussian even at the longest diffusion time probed in this experiment. Given thecomposition of the water spectroscopic voxel used (nearly totally GM), we are also confidentthat the presence of multiple T_1_pools, or exchange between multiple T_1_pools during TM, is very unlikely to bias our diffusion measurements (Fig. S1). Furthermore, by plotting logS(*b*= 0) = f(TM) (not reported), TM being the mixing time, wedid not observe multiple T_1_pools that could bias the signal, or diffusivityestimates as a function of*t_d_*.

The main difference from previous results (Jelescu et al.,2022;Lee et al., 2020) is in the D_W_time-dependence. On the one hand, we measured a significant decrease in D_W_(from0.62 to 0.57 µm²/ms) over the*t_d_*range 20-42.5 ms,while on the other handJelescu et al. (2022)andLee et al. (2020)report an almost constant D_W_over this same*t_d_*range in rat cortex and hippocampus and over a*t_d_*range 20–100 ms in human cortical GM, respectively. Oncloser inspection, the ~0.05–µm²/ms difference measured in our study iswithin the range of inter-animal variability reported in these other studies, which mayexplain the lack of significant difference in these other studies.

A decrease in D_W_and K_W_over the entire*t_d_*range suggests a nontrivial influence of microstructure, such as structural irregularities,and therefore the diffusion cannot be considered Gaussian, even at the longest diffusion timemeasured in this experiment. Moreover, these observations may also indicate an influence ofthe exchange between the intracellular and extracellular spaces, or a contribution of the twophenomena, structural disorder and exchange.

#### 3.1.3 Structural disorder

According to the literature (Lee et al., 2020;Novikov et al., 2014), intra-compartmental diffusion can beinfluenced by structural disorder, and thus the assumption of Gaussian diffusion is broken. Inparticular, works based on the coarse-graining approach have shown thatD_W_(*t_d_*) andK_W_(*t_d_*) can reflect different classes of structuraldisorder in 1D, 2D, or 3D (Lee et al., 2020;Novikov et al., 2014). Therefore, we compared differentfunctional forms to fit the data and identify which structural disorder might dominate thewater behavior.

**Fig. 2.**
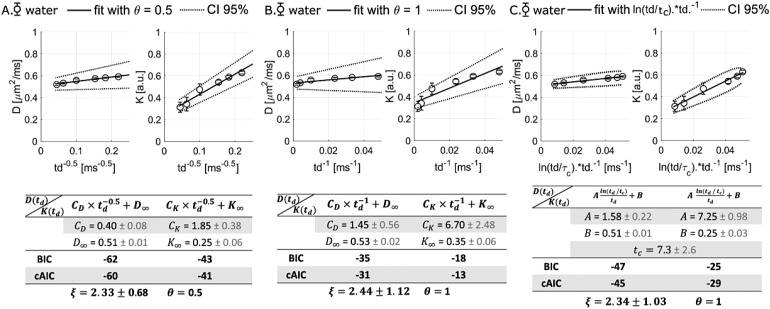
D_W_(*t_d_*) andK_W_(*t_d_*) averaged over four mice and differentfunctional forms fitted to these data. (A) The 1D structural disorder form is correctlyfitted to D and K. (B) The 2D structural disorder form does not fit well to the data, withthe highest AIC for D and K. (C) The 2D functional form in the limit of θ =(p+d)/2 = 1 fits the data with a cAIC close to the cAIC estimate for the 1Dstructural disorder form. The dimensionless ratio$\xi =C_{K}/(C_{D}/D_{\infty })$~ 2 whatever the functional forms fitted to these data, which iscompatible with pure 1D structural disorder (ξ = 2).

First, three models were applied to determine whether 1D or 2D/3D structural disorder coulddominate. Based onLee et al. (2020)andNovikov et al. (2014), we consider models given by

$$D(t_{d})=D_{\infty }+C_{D}t_{d}^{-\theta }$$ [2]

$$K(t_{d})=K_{\infty }+C_{K}t_{d}^{-\theta }$$ [3],

with the constants C_D_, C_K_,$D_{\infty }=\lim_{t_{d}\to \infty }D$and$K_{\infty }=\lim_{t_{d}\to \infty }K$left as free parameters to be determined by fitting the experimental dataand setting the “universal” dynamical exponent θ = (p+d)/2to 0.5 (representing 1D structural disorder with a dimensionality d = 1 and astructural exponent p = 0, which determines the structural universality class) and 1(representing either 2D/3D structural disorder, with either p = 0 and d = 2corresponding, for example, to random disks or p = -1 or d = 3 corresponding,for example, to random rods), respectively (Fig. 2A-B).In gray matter, neuronal and glial cell soma, represented as spherical objects with~5–8-µm radii, can also induce a 1/t power law decay ofD_W_(*t_d_*)/K_W_(*t_d_*)for long diffusion times*t_d_*>>R²/D_0_. If water probes an extracellular space mostly comprising randomlyoriented fibers, D_W_(*t_d_*) andK_W_(*t_d_*) may also exhibit a logarithmic singularity ofthe form ln(*t_d_*/tc)/*t_d_*at long*t_d_*(Burcaw et al., 2015).Hence, we also consider the alternative model

$$\text{A }\left(\text{ln(}t_{d}/\text{tc)}\right)/t_{d}+\text{B},$$ [4]

with A a coefficient proportional to the universal dimensionless tail ratio, and B acoefficient corresponding to the long time limit (Fig.2C).

Visually, it is not clear whether D_W_and K_W_follow a power law withexponent θ = 0.5, θ = 1 orequation [4]. The results of the fit with the specific 1D structural disorder formare not too different from the results of the fit withln(*t_d_*/tc)/*t_d_*, according to cAIC thatis low for both models. In addition, it is not clear that D_W_and K_W_follow exactly the same law. At this stage, it is therefore difficult to determine whether oneor the other form of structural disorder dominates.

**Fig. 3.**
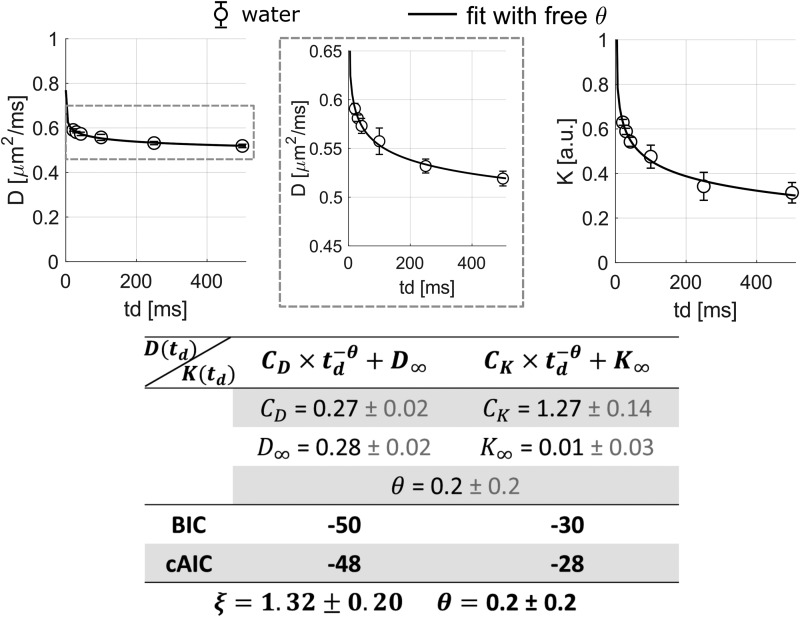
D_W_(*t_d_*) andK_W_(*t_d_*) averaged over four mice and fit withstructural disorder model. Joint fit of a power-law decay with a free exponent theta toD_W_and K_W_, with the same free exponent theta.

To go further, a model with exponent θ inequation[2]andequation [3]left as an additionalfree parameter to be determined from the data was used to fit D_W_and K_W_(Fig. 3). The fit gives θ = 0.2 ±0.2 (which is closer to 0.5 than 1), and the universal dimensionless tail ratio$\xi =C_{K}/(C_{D}/D_{\infty })=1.3\pm 0.2$(which is close to 2). Values of θ ~0.5 and ~2 are expected for 1Dshort-range structural disorder (Lee et al., 2020;Novikov et al., 2014). We also found a tail ratio of~2 regardless of the model used for fitting, with a best precision. Fit with free exponent isless accurate because of the small number of*t_d_*.

### 3.2 Unique t _d_ dependence of metabolite diffusion in the intracellular space

#### 3.2.1 Methodological considerations

**Fig. 4.**
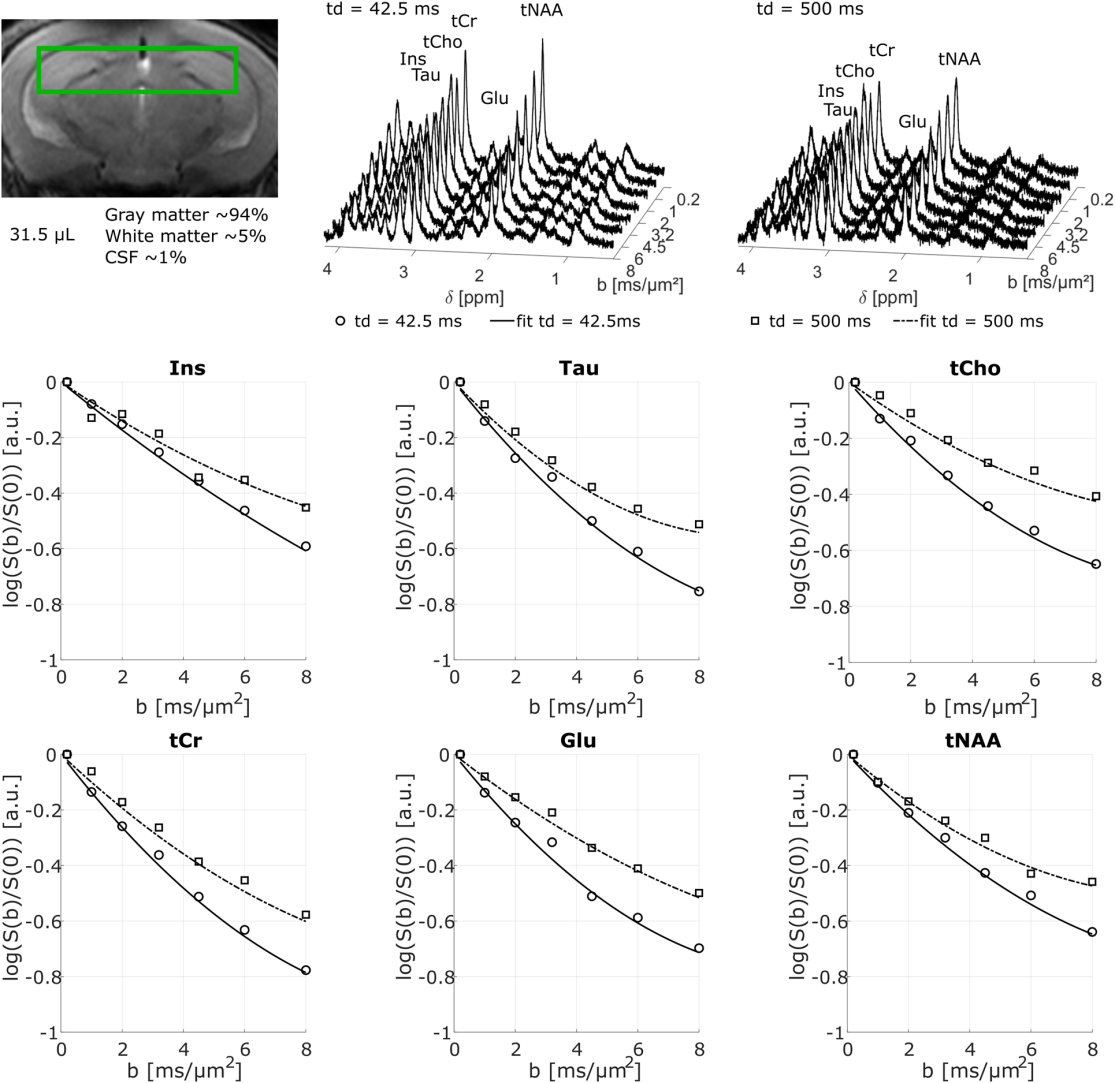
Example of spectra and attenuation curves for each metabolite as a function ofdiffusion-weighting*b*acquired on one mouse for two different diffusiontimes in a voxel around hippocampus (image).

Spectra acquired in the hippocampal voxel have a good signal-to-noise ratio (>20 forNAA for the longest*t_d_*and highest*b*value) evenat the longest diffusion times and highest*b*values (Fig. 4) and the LCModel analysis (not shown here) is robust (CRLB ~2% for allmetabolites even at the longest diffusion time and highest*b*value) and wefind a correct estimate of S/S0 with low inter-animal variability. The model given byequation 1also fits the data well for each metabolite andanimal (Fig. 4).

#### 3.2.2 D _M_ decreases and K _M_ increases

**Fig. 5.**
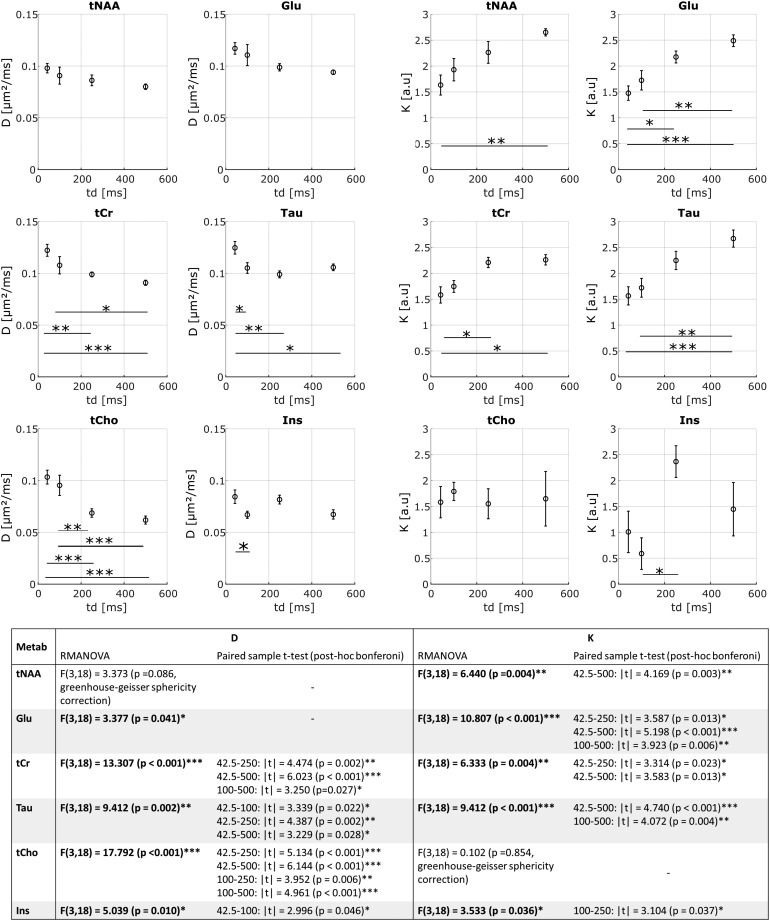
D_M_(left panel) and K_M_(right panel) averaged over seven mice as afunction of diffusion time. For each metabolite, D_M_decreases and K_M_increases.

For each metabolite, with the exception of Ins, the inter-animal estimate of D_M_and K_M_is relatively consistent between animals with a standard error of the meanof less than 9% for D_M_and 30% for K_M_(Fig. 5). D_M_decreases to a plateau of ~0.08 µm²/ms for NAA,Glu, tCr, and Tau, and ~0.06 µm²/ms for tCho and Ins with increasing*t_d_*for all metabolites, and the D_M_estimate isclearly in agreement with previous results (Palombo et al.,2016). Despite the low number of*t_d_*, imposed by an in vivoexperimental setup, statistical analysis with a paired-sample t-test shows a significantincrease in K_M_and decrease in D_M_for each metabolite, with at least apower of 0.7 for our sample size N = 7. The diffusivity of predominantly intra-glialmetabolites (Fisher et al., 2002) is lower than that ofother metabolites. These differences are also noticeable in the estimate of K_M_,which increases with*t_d_*for NAA, Glu, tCr, and Tau and tendsalmost to a plateau ~2.3–2.5 while Ins and tCho remain almost constant ~1.5.

Such an increase in K is expected for diffusion in a restricted environment and thusstrongly affected by the microstructure, in agreement with the numerical simulations ofIanus et al. (2021), which account for restriction in soma,exchange between soma, and cellular processes and cellular processes branching. Thedifferences on the diffusion parameters observed between glial and neuronal cells aretherefore not surprising because of the rather different morphology of these two cell types(Palombo, Shemesh, et al., 2018). Besides the highsensitivity of these dMRS methods for probing microstructure, already reported in previousstudies (Ligneul et al., 2019), this work shows thateven at lower*b*values (≲10 ms/µm²), suitable for thevalidity range of the kurtosis representation, different cell microstructures can bedistinguished. dMRS thus appears a powerful tool to probe the cell morphology with potentialfeasible translation to*in vivo*human measurements, using also clinicalscanners with relatively low maximum gradient strength (~60-80 mT/m).

#### 3.2.3 Intracellular metabolites as endogenous probe of the intracellular microarchitecture

**Fig. 6.**
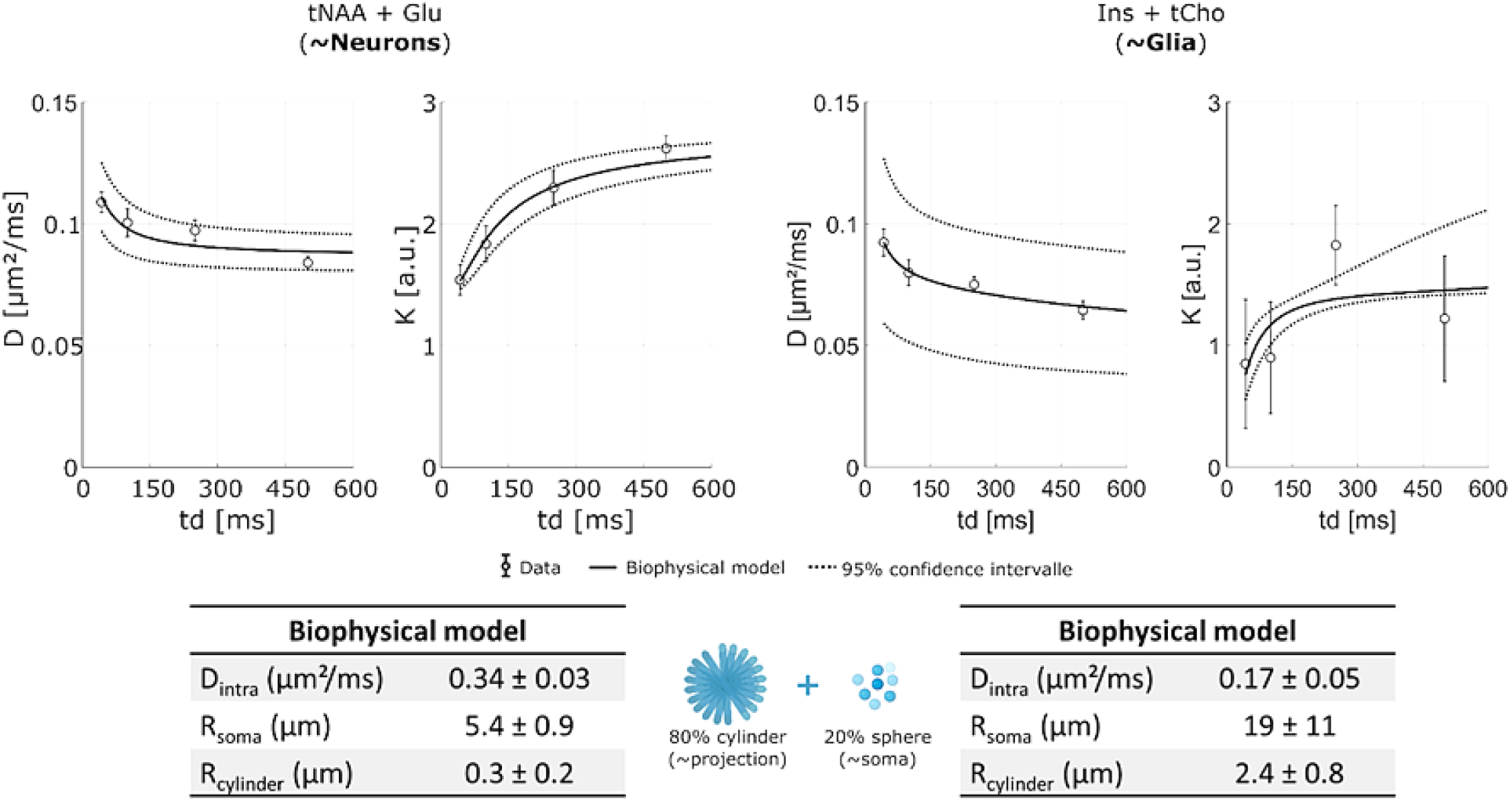
Fitting of a biophysical model to predominantly intraglial (upper panel) or preferentiallyintraneuronal (lower panel) metabolite data. The biophysical model is a very simple modelcomposed of 80% isotropically distributed cylinders and 20% spheres, representingprojections and soma, respectively.

To go further, we therefore proposed to evaluate the influence of the restricted environmenton diffusion by fitting to D_M_(*t_d_*) andK_M_(*t_d_*) a simple model representing the intracellularcompartment through the soma and projections formed by 20% spheres and 80% randomly orientedcylinders. Due to the low number of measurements, this ratio is imposed to avoid overfittingthe model. Using an in-house iterative algorithm, the model was fitted to the D_M_and K_M_of mostly intra-neuronal (NAA and Glu) and predominantly intra-glial (tChoand Ins) metabolites (Fisher et al., 2002;Gill et al., 1989;Griffinet al., 2002;Urenjak et al., 1993), to extractradii from the soma and projections of (hypothetical) neuronal and glial cells, respectively(Fig. 6). First, this restriction model appears to besuitable for describing the behavior of D_M_(*t_d_*) andK_M_(*t_d_*). For neuronal cells, this model gives estimateswith low standard error of the radii of spheres andD_M_(*t_d_*= 0), and the estimates are consistentwith previous results, whereas the radii of cylinders are rather poorly estimated. Forintra-glial metabolites, the uncertainty in the sphere and cylinder radii is high and theestimate is overestimated, but the D_M_(*t_d_*= 0) isconsistent with the literature in normal mouse GM (Ligneul etal., 2019;Palombo et al., 2020). Of course,the aim of this work is not to propose a new model for estimating biophysical parameters, butthese results suggest that even such a simple model, like SANDI (Palombo et al., 2020), describes metabolites data well.

In conclusion, the*t_d_*-dependent diffusion of intracellularmetabolites is dominated by restriction in the intracellular space, in line with a previousstudy with various dMRS methods (Assaf & Cohen,1999;Kroenke et al., 2004;Ligneul et al., 2017;Shemesh et al.,2014,^2017^;Valette et al., 2018;Vincent et al., 2020)and it is not surprising that it is clearly influenced by cell morphology (cell projectionsize and soma size at least). Importantly, as K_M_does not decrease at increasing*t_d_*, no signature of 1D structural disorder along the neurites isobserved for the metabolites, in contrast with what has been speculated for intracellularwater. The*t_d_*dependence of metabolite diffusion seems thusclearly more specific to cell morphology than water.

### 3.3 Intracellular metabolite diffusion to highlight the role of exchange in water diffusion time-dependence

It is insightful to examine water and metabolites results in the light of the work ofAggarwal et al. (2020). Our experimental results aboutintracellular metabolite kurtosis time-dependence, that is, kurtosis increasing with*t_d_*, are in line with the behavior simulated for long exchangetimes inAggarwal et al. (2020). In contrast, waterkurtosis time-dependence exhibits the opposite behavior, which is in line with the behaviorexpected for a system with significant exchange inAggarwal etal. (2020). This strongly suggests fast water exchange relative to the diffusion timesused in the present study, which is in agreement with previous works suggesting short exchangetime (Jelescu et al., 2022;Lee et al., 2020). The influence of such fast exchanges is alsosuggested in our estimate of the free exponent, θ = 0.2, found inFigure 3, which is close to that found by Jelescu et al. whenthey fitted their simulated exchange-driven K for a short exchange time ~25 ms, with the samepower-law decay with a free theta exponent.

**Fig. 7.**
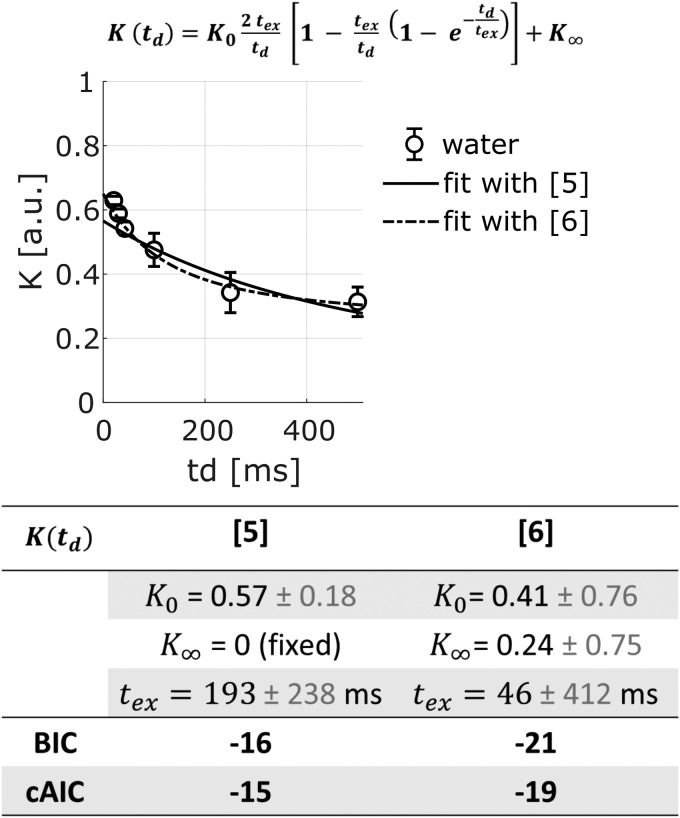
Fit of the Kärger model (equation [5]) andthe constant-modified Kärger model (equation[6]) to the K_W_data for*t_d_*∈ [42.5; 500ms], where D_W_(*t_d_*) is nearly constant.

**Fig. 8.**
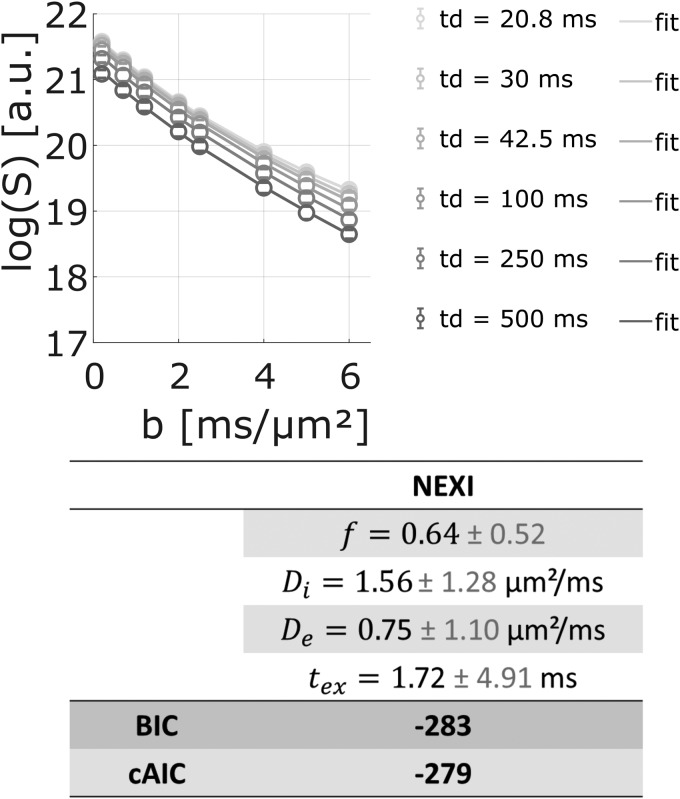
Fit of the water signal (log(S)) as a function of*b*up to 6ms/µm² with the NEXI/SMEX model.

To provide a quantitative estimate of exchange time for our experimental conditions, we fitan increasingly used (Fieremans et al., 2010;Jelescu et al., 2022;Jensen,2023;C. Li et al., 2023;Moutal et al., 2018;Olesen et al.,2022;Zhang et al., 2021) model of exchange: theKärger Model

$$K\left(t_{d}\right)=K_{0}\frac{2t_{ex}}{t_{d}}\left[1-\frac{t_{ex}}{t_{d}}\left(1-e^{-\frac{t_{d}}{t_{ex}}}\right)\right]$$ [5]

$$K\left(t_{d}\right)=K_{0}\frac{2t_{ex}}{t_{d}}\left[1-\frac{t_{ex}}{t_{d}}\left(1-e^{-\frac{t_{d}}{t_{ex}}}\right)\right]+K_{\infty }$$ [6]

to the data for the longest diffusion time [42.5; 500] ms, to satisfy one of the assumptionsof the Kärger model, that is, constant D_W_(*t_d_*)(Fig. 7). The estimated exchange time is very impreciseand spans hundreds of ms: from 46 (±412) ms (usingequation [6]) to 196 (±238) ms (usingequation[5]). Regardless of the poor precision despite high data quality, the contradictionwith the previous estimate of exchange time shorter than ~25 ms (as discussed in the previousparagraph) might be due to the fact that the assumptions behind the Kärger Model are notsatisfied. According toFieremans et al. (2010), thenon-applicability of the Kärger Model suggests that the membrane between theintracellular and extracellular spaces is too permeable to allow for an accurate descriptionusing the Kärger Model. Other recently proposed models of exchange are NEXI (Jelescu et al., 2022) and SMEX (Olesen et al., 2022); both based on the same modified KärgerModel to account for potential exchange between neurites and extracellular space. FittingNEXI/SMEX to the water data as a function of*b*value (for extra*b*values up to 6 ms/µm²) and diffusion time (Fig. 8) yields once more very imprecise estimates of exchangetime: 1.7 (±4.9) ms (other model parameters estimates are: intraneurite diffusivity 1.56± 1.28 µm²/ms; extraneurite diffusivity 0.75 ± 1.10µm²/ms and neurite signal fraction 0.64 ± 0.52). Although imprecise, theNEXI/SMEX estimate of exchange time is more in agreement with the evidence of exchange timeshorter than 10 ms discussed in the previous paragraph and supported by recent works (Cai et al., 2022;Olesen etal., 2022;Williamson et al., 2019,^2020^,^2023^).

In conclusion, these spectroscopic measurements with lower variability and over a wider rangeof*t_d_*than the previous studies (Jelescu et al., 2022;Olesen et al., 2022)corroborate the influence of exchange on water diffusion as a function of time (in vivo in themouse GM). The comparison of the very different functional forms of the measuredD_W_(*t_d_*)-K_W_(*t_d_*)and purely intracellularD_M_(*t_d_*)-K_M_(*t_d_*)supports the hypothesis that the signature of 1D or 2D/3D short range structural disorderobserved forD_W_(*t_d_*)-K_W_(*t_d_*)might mostly come from the water diffusion in the extracellular space, which“contaminates” the whole water pool signature due to fast exchange and hencehides intracellular signature. Our measurements reinforce the need for a model describing the*t_d_*-dependence of diffusion in GM, with specific signatures inintra- and extracellular compartments and inter-compartmental exchange, to precisely evaluatehow exchange can mask the signatures of intracellular space.

## 4 Limitations

The range of*t_d_*values over which we were able to carry out ourmeasurements remains rather restricted, even if it is the widest ever probed in this type of*in vivo*measurement. On the one hand, a constraint linked to the duration ofthe spoiling gradient applied during the mixing time in this sequence (Ligneul et al., 2017) prevents us from having access to smaller*t_d_*values. With a shorter*t_d_*, we couldobserve the inflection point of the time-dependent kurtosis of water, which could help determinethe average permeability. Future work could try to combine OGSE and PGSE measurements to try tocharacterize the time-dependence at shorter*t_d_*. On the other hand,the increase in*t_d_*is limited by the SNR, which becomes too lowbeyond 500 ms. Among other things, this increase could allow us to better discriminate whichclass of structural disorder dominates, which would be a first step towards disentangling thedifferent contributions of exchange and structural disorder.

Our aim here is not to propose a new modeling method, but to present experimental data thatcould support theoretical models to better describe gray matter. We have chosen to placeourselves in a diffusion regime suited to a representation with the cumulant exponent, whichseems quite sensitive to exchange. Nevertheless, with our experiments we were not able toprecisely determine the exchange time in gray matter (using currently available biophysicalmodels, that is, Kärger model and NEXI/SMEX), and to determine the influence ofstructural disorder on the water diffusion properties. The complexity of both exchangemechanisms (e.g., multiple exchange times) (Nilsson et al.,2013) and microstructure (e.g., glial and neuronal cells and complex extracellularspace) (Palombo et al., 2020) will need to be taken intoaccount in future work to disentangle the contributions of exchanges and structural disorder.Towards this goal, future work could harness advanced computational modeling tools for graymatter microstructure simulations (Callaghan et al.,2020;Palombo et al., 2016,^2020^).

We also chose a smaller voxel size for water, in order to focus only on gray matter withoutmultiple compartment effects that can complicate interpretation of the*t_d_*dependence of the diffusion and to be in conditions similar tothose encountered in the literature for segmented images of rodent brain (Jelescu et al., 2022). In particular, in this voxel, we minimize thecontribution of CSF and white matter. For metabolites, we have no choice to take this largevoxel to have sufficient signal at long diffusion time; however, the contribution of CSF and WMremains small (respectively ~5% and 1%), which can certainly have a negligible impact on ourresults. Indeed, we did not notice a strong signal attenuation at low*b*whichwould have suggested a high CSF contribution whatever the metabolite. This is in line withmeasurements carried out byLundell et al. (2021)in thehuman brain, where they noted that for a CSF contribution of ~2% there was no visible effect ondiffusion, whereas for a contribution of ~10% there was. Finally, by fitting a bi-exponentialmodel to log(S0) as a function of mixing time, only a small fraction of the short T_1_component was estimated, suggesting that signal is not affected by multi-T_1_pool, andcertainly not influenced by short T_1_of WM (seeSupplementary Materials, Figs. S1 andS2).

In this work, we focused on healthy mice; future work will explore the ability of combinedD_W/M_(*t_d_*)-K_W/M_(*t_d_*)to characterize pathological microstructural changes. Moreover, it could be informative toextend in future work the investigation ofD_M_(*t_d_*)-K_M_(*t_d_*) toalso metabolites that are in both intra- and extracellular space and potentially in exchange.Further extension of this work will include measurements in humans to assess the feasibility andtranslatability of this approach.

## 5 Conclusion

This experimental study proposes for the first time a comparison of the*t_d_*dependence of the diffusion of two different endogenous probes,water and intracellular metabolites. The information provided by these two sets of*t_d_*-dependent acquisitions is complementary, especially since theyprobe different compartments of the GM. In particular, the*t_d_*dependence of water diffusion is mainly dominated by short-range structural disorder in theextracellular space and rapid exchanges between intra- and extracellular spaces, whereas the*t_d_*dependence of intracellular metabolite diffusion is morespecific to restriction within the intracellular space. Other underlying features could befurther investigated to fully decipher the sources contributing to total kurtosis (beading,undulation,…), as already proposed by Shemesh Lab (Alveset al., 2022;Henriques et al., 2020).Nevertheless, these new results help interpretD(*t_d_*)-K(*t_d_*) measurements in terms ofthe underlying microstructure of GM and suggest thatD(*t_d_*)-K(*t_d_*) measurements ofmetabolites may offer a new way to quantify microstructural restrictions in GM.

## Supplementary Material

Supplementary Material

## Data Availability

All the data and analysis codes underpinning the results presented here can be found in theCardiff University data catalogue at thehttp://doi.org/10.17035/d.2024.0314230392.
