# Supplementary material for: Investigating exchange, structural disorder, and restriction in gray matter via water and metabolites diffusivity and kurtosis time-dependence
Source: Imaging Neurosci (Camb). 2024 Apr 5;2:imag-2-00123. doi: 10.1162/imag_a_00123 (PMC12247622; doi:10.1162/imag_a_00123)
Supplement: Supplementary Material [file imag_a_00123-supp.pdf]

SUPPLEMENTARY MATERIAL for "Investigating exchange, structural disorder and restriction in Gray Matter via water and metabolites diffusivity and kurtosis time-dependence"

The presence of multiple T_1 pools could bias the results of stimulated echo (STE), with a bigger time-dependence than that measured by the spin-echo sequence (Lee et al., 2017).

To address this question for water and metabolite, the logarithm of the "true" S0 signal (as extracted when fitting the kurtosis representation) was plotted as a function of the mixing time (TM) (fig.S1 and fig.S2 respectively) and fitted with the two equations below, which describe signal when TR is not long compared to T_1 (i.e. longitudinal magnetization is not fully relaxed), for either one T_1 pool or two (short and long) T_1 pools:

$$S(TM) = S0\,(1 - exp(-(TR - TM)/T_1)\,) \times exp(-(TM)/T_1) \qquad \text{eq.[S1]}$$

$$S(TM) = S0\,f_{short}\,(1 - exp(-(TR - TM)/T_{1short})\,) \times exp(-(TM)/T_{1short})$$

$$+ (1 - f_{short})(1 - exp(-(TR - TM)/T_{1long})\,) \times exp(-(TM)/T_{1long}) \qquad \text{eq.[S2]}$$

where a single T_1 or two different T_1 (short and long) are considered.

Results suggest that a single T_1 pool exists for two cases.

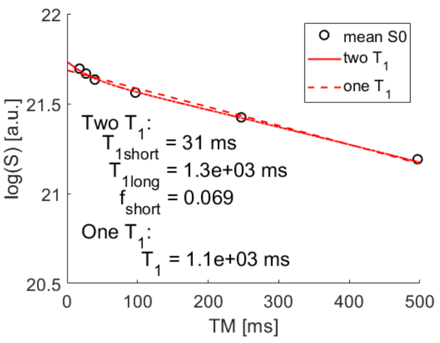

Fig.S1: log(S0) = f(TM) for water, with the averaged S0 extracted from fitting the kurtosis representation. The fit using eq.[S1] or eq.[S2] is represented by a dotted line and a solid line. The short T_1 fraction pool is really small (<7%), suggesting a single T_1 pool.

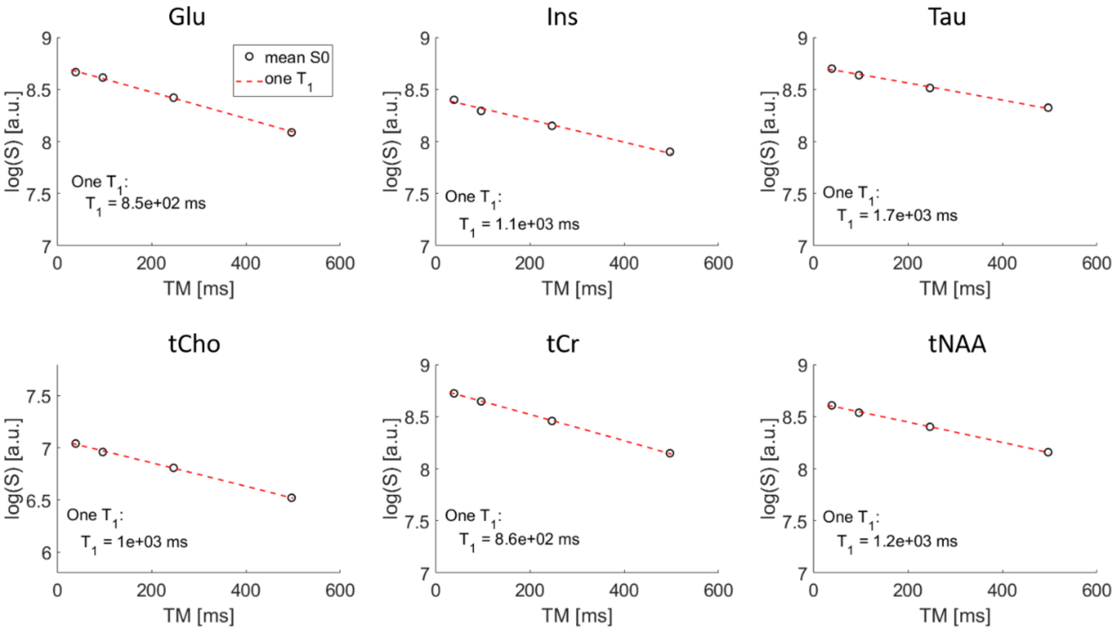

Fig.S2: log(S0) = f(TM) for each metabolite with the averaged S0 extracted from fitting the kurtosis representation. The fit using eq.[S1] is represented by a dotted line. The two-pool model overfits the data, therefore it is not shown here.

Reference

Lee, H.-H., Novikov, D., & Fieremans, E. (2017). T1-induced apparent time dependence of diffusion coefficient measured with stimulated echo due to exchange with myelin water. *Proc. Intl. Soc. Mag. Reson. Med. 25*, 0839. https://index.mirasmart.com/ISMRM2021/PDFfiles/2199.html
